# *Helicobacter pylori* Secreted Protein HP1286 Triggers Apoptosis in Macrophages via TNF-Independent and ERK MAPK-Dependent Pathways

**DOI:** 10.3389/fcimb.2017.00058

**Published:** 2017-02-28

**Authors:** Raquel Tavares, Sushil Kumar Pathak

**Affiliations:** Department of Molecular Biosciences, The Wenner-Gren Institute, Stockholm UniversityStockholm, Sweden

**Keywords:** Helicobacter, apoptosis, macrophages, secreted proteins, MAPKs

## Abstract

Macrophages constitute a powerful line of defense against *H. pylori*. The final disease outcome is highly dependent on the bacterial ability to modulate the effector functions of activated macrophages. Here, we report that *H. pylori* secreted protein HP1286 is a novel regulator of macrophage responses. Differential expression and release of HP1286 homologues were observed among *H. pylori* strains. Recombinant purified HP1286 (rHP1286) had the ability to bind to primary human monocyte-derived macrophages (MDM) and macrophage cell lines. Exposure to rHP1286 induced apoptosis in macrophages in a dose- and time-dependent manner. Although interaction of rHP1286 was observed for several other cell types, such as human monocytes, differentiated neutrophil-like HL60 cells, and the T lymphocyte Jurkat cell line, rHP1286 failed to induce apoptosis under similar conditions, indicating a macrophage-specific effect of the protein. A mutant strain of *H. pylori* lacking HP1286 protein expression was significantly impaired in its ability to induce apoptosis in macrophages. Significantly higher caspase 3 activity was detected in rHP1286-challenged macrophages. Furthermore, rHP1286-induced macrophages apoptosis was not inhibited in the presence of neutralizing antibodies against TNF. These observations indicate that rHP1286 induced a caspase-dependent and TNF-independent macrophage apoptosis. Pre-treatment of macrophages with U0126, an inhibitor of the ERK MAPK signaling pathway significantly reduced rHP1286-induced apoptosis. Furthermore, nuclear translocation of ERK and phosphorylation of c-Fos was detected in rHP1286-treated macrophages. These results provide functional insight into the potential role of HP1286 during *H. pylori* infection. Considering the ability of HP1286 to induce macrophage apoptosis, the protein could possibly help in the bacterial escape from the activated macrophages and persistence in the stomach.

## Introduction

The Gram-negative bacterium *Helicobacter pylori* is known to selectively colonize the human gastric mucosa for over 50,000 years. Currently, more than half of the world's population is colonized by *H. pylori*. Even though most of the infections are asymptomatic, long-term colonization can lead to chronic gastritis and the development of ulcers, adenocarcinoma and mucosa-associated lymphoid tissue (MALT) lymphoma (Wroblewski et al., 2010). In spite of the generation of strong immune responses against the bacterium, the host fails to eradicate the infection in the majority of cases. This is partly due to the fact that the bacterium has developed several strategies to modulate the effector functions of immune cells for its own survival and benefit. Macrophages play an important role in the host defense against *H. pylori* through several strategies, such as phagocytosis, production of various cytokines/chemokines, and microbicidal compounds such as ROS and NO (Wilson et al., 1996; Gobert et al., 2002a,b). However, *H. pylori* is known to block or regulate these macrophage strategies, which results in inefficient bacterial killing. Induction of programmed cell death or apoptosis in macrophages is a well-known bacterial strategy that helps in the colonization and persistence of the bacterium. It has been reported previously that *H. pylori* or bacterium-derived products induce apoptosis in macrophages, which occurs via polyamine-dependent mechanisms and signaling via ERK-MAPK and the Src family of tyrosine kinases (Allen et al., 2000; Zheng and Jones, 2003; Chaturvedi et al., 2004; Asim et al., 2010; Pathak et al., 2013).

Despite the small size of the *H. pylori* genome, a large fraction, presumably from 30 to 40%, is annotated as hypothetical proteins with unknown function (Zanotti and Cendron, 2014). Among this group, many are secreted by the bacterium. Based on various studies, the *H. pylori* secretome appears to be composed of approximately 160 proteins (Zanotti and Cendron, 2014). Considering the general non-invasive nature of *H. pylori*, it is believed that secreted proteins play an important role in the bacterial survival and ultimate disease development. The potential role of some secreted proteins, such as Vacuolating cytotoxin A (Vac A), Tumor necrosis factor alpha-inducing protein (Tip-alpha), HP0175, HP0305, and Neutrophil-activating protein (HP-NAP) has been suggested in various studies (Suganuma et al., 2005; D'Elios et al., 2007; Palframan et al., 2012; Pathak et al., 2013). However, there are still many other hypothetical or less-studied secreted proteins of *H. pylori* whose role in bacterial pathogenesis remains unidentified. With this in view, this study was focused on functional characterization of the protein HP1286.

Presence of HP1286 in the external medium of *H. pylori* culture has been reported in several independent studies (Bumann, 2002; Kim et al., 2002; Müller et al., 2013). Crystal structure analysis led to the placement of HP1286 in the family of YceI-like proteins, due to the presence of a cavity formed by an eight-stranded β-barrel (Sisinni et al., 2010). However, based on the structure and shape of the internal cavity, which varies from other members of the family, it was suggested that HP1286 has the function of binding and/or transporting amphiphilic molecules (Sisinni et al., 2010). Another study on the adaption of *H. pylori* to acidic stress reported that HP1286 expression is strongly up regulated in a UreI-negative strain, a mutant unable to transport urea inside the cell (Toledo et al., 2002). In addition, a recent study showed that recombinant HP1286 induces apoptosis in gastric epithelial cell line AGS (Li et al., 2012). The balance between cell death and cell growth is essential for the normal function of gastric mucosa. By induction of apoptosis in gastric epithelial cells, HP1286 along with other known apoptosis-inducing factors of *H. pylori* (Kuck et al., 2001; Basak et al., 2005; Kim et al., 2007) could eventually damage the gastric epithelial cell layer, allowing the interaction of HP1286 and other virulence factors with various immune cells in the lamina propria.

In this study, we have studied the apoptosis-inducing ability of HP1286 on other possible target cells such as macrophages, monocytes, neutrophils, and T cells. We provide evidence indicating that although rHP1286 interacts with several immune cells, it is capable of inducing apoptosis in only macrophages. Apoptosis inducing ability of a *H. pylori* mutant strain 26695, lacking HP1286 expression, was significantly impaired. In addition, we have identified the host cell signaling pathways that regulate rHP1286-induced apoptosis in macrophages.

## Materials and methods

### Bacterial strains and culture conditions

*H. pylori* strains 26695 (ATCC 700392D-5), J99 (ATCC 700392), TN2GF4, P12, CCUG17875, HPAG1, 67:21, and 67:20 has been described previously (Björkholm et al., 2001; Basmarke-Wehelie et al., 2011; Tavares and Pathak, 2015). The bacteria were grown on Columbia blood agar plates (Acumedia) supplemented with 8% horse blood (Hatunalab) and 8% horse serum (Hatunalab) at 37°C under microaerophilic conditions. For growth in liquid culture, cells were grown in Brucella broth (Acumedia) containing 8% horse serum with shaking at 37°C under microaerophilic conditions. The *Escherichia coli* BL21 (DE3) and DH5α strains were grown in Luria-Bertani Miller (LBM) media.

### Cell culture and treatments

All cell lines were obtained from American Type Culture Collection except HEK293-hTLR4A-MD2-CD14 (HEK-TLR4) cells (InvivoGen). The murine macrophage cell line RAW264.7 (TIB-71) was cultured in DMEM (Gibco, Life Technologies) supplemented with 10% heat-inactivated fetal bovine serum (FBS) (Sigma-Aldrich). Human monocytic cell line THP-1 (TIB-202), human T lymphocyte cell line Jurkat (TIB-152), and human HL-60 promyelocytic leukemia cells were maintained in RPMI-1640 (Gibco, Life Technologies) with 10% FBS at 37°C and 5% CO_2_. THP-1 cells were differentiated into macrophages in the presence of 100 nM phorbol 12-myristate 13-acetate (PMA) (Sigma-Aldrich) for 3 days. HL-60 cells were treated with 1 mM Retinoic acid (RA) (Sigma-Aldrich) for 7 days to differentiate into neutrophil-like cells. HEK-TLR4 cells were cultured in DMEM with 10% FBS, 50 U/ml penicillin, 50 μg/ml streptomycin, 2 mM l-glutamine, 10 μg/ml Blasticidin (InvivoGen), and 50 μg/ml HygroGold (InvivoGen). As indicated, cells were treated either with various concentrations of rHP1286 or the same volume of protein storage buffer. As a positive control, *E. coli* LPS (500 ng/ml; Sigma-Aldrich) was used. In some experiments, cells were pretreated with vehicle alone or U0126 or ZVAD-FMK (Calbiochem, Merck4biosciences) for 30–60 min. The neutralizing TNF antibody (10 μg/ml, clone MP6-XT22; MAB4101) was obtained from R&D Systems. For infection studies, wild-type *H. pylori* strain 26695 (Wt) or *hp1286*-deficient mutant strain (Δ*hp1286*) was added to macrophages at MOI100. For the preparation of *H. pylori* conditioned medium (CM), Wt and Δ*hp1286* strains were grown in DMEM supplemented with 10% FBS and 0.5% yeast extract for 20 h. CM was filtered with a 0.22 μm filter and added to RAW264.7 cells at 1:4 ratio.

### *In vitro* differentiation of MDMs

MDM were generated as described previously (Pathak et al., 2013). Briefly, RosetteSep human monocyte enrichment cocktail (Stem Cell Technologies) was used to isolate CD14-positive monocytes from buffy coats (obtained from the blood bank at Karolinska University Hospital, Huddinge) by negative selection. Isolated cells were stained with anti-CD14 antibody (Clone TUK4; DAKO) to determine the purity. Monocytes were allowed to adhere on cell culture plates for 2 h followed by washing of unbound cells. Monocytes were cultured for 6–7 days in RPMI 1640 medium supplemented with 10% FBS and recombinant human macrophage colony-stimulating factor (M-CSF; 50 ng/ml; Immunotools). To assess the differentiation of monocytes into macrophages, changes in cell morphology were monitored by light microscopy (Carl Zeiss, Axiovert 40 C) and cells were stained with anti-CD68 (clone Y1/82A; BD biosciences) and anti-CD14 antibodies. Sample acquisition was performed using LSRFortessa flow cytometer (BD Biosciences).

### Generation of *H. pylori* 26695Δ*hp1286* mutant strain

Genomic DNA from *H. pylori* 26695 was isolated using a Genomic DNA isolation kit (Promega) following the manufacturer's instructions. *hp1286*-specific primers A (5′-TCTAAAAAGTCTAGCGCTCCGGGAA-3′), B (5′-CCAACTTACTGATTTAAACGCCAAACTAACACCAA-3′), C (5′- AATAAGTGATAATAAGGAGATGAGGTAAAGATTGAG-3′), D (5′-AGAGGACTTCAAATCACTCAAAAAGAATTGC-3′), were used to amplify the up- and downstream regions, respectively. The Chloramphenicol resistance gene (CAT) was amplified by PCR from pACY184 vector using primer pair Cat 1(5′- GTTAGTTTGGCGTTTAAATCAGTAAGTTGGCAGCAT-3′) and Cat 2 (5′- CTCAATCTTTACCTCTTATTATCACTTATTCAGGCG -3′). PCR products were purified using the Gel purification kit (OMEGA bio-tek). The primers were designed with overlapping homologies at the end. PCR reaction was performed using three PCR products for five cycles without any primers followed by 30 cycles with primers A and D. The purified PCR product was mixed with *H. pylori* strain 26695 to allow natural transformation using the method of spot transformation. 150 ng of purified PCR product was mixed with 100 μl of bacteria (1 × 10^8^ bacteria/ml stock) and spotted onto blood agar plates. After 6 h, the transformation spot was spread over the plate and incubated for further 24 h. The bacteria were then moved onto selective blood agar plates containing 15–20 μg/ml of Chloramphenicol and incubated for 3 to 7 days. Positive clones were identified by PCR. Absence of HP1286 expression in the mutant strain was confirmed by western blot using an antibody raised against rHP1286.

### Cloning, expression, and purification of HP1286

The construct for expression of sequence encoding amino acids 18 to 182 of HP1286 was generated by cloning of the PCR product at NdeI and XhoI sites of the vector pET28b+ (Novagen). The genomic DNA from *H. pylori* 26695 was used as template in the PCR reaction. Primers used were: 5′-GCGAGCTCATATGAAACCTTATACGATTG-3′ (Forward) and 5′-AAACTCGAGTTGGGCGTAAGCTTCTA-3′ (Reverse). Induction of the protein was carried in *E. coli* BL21 (DE3) at 37°C for 3–4 h with the addition of isopropyl thio-β-d-galactoside (100 μM) in Luria-Bertani (LB) media. Hexa-His-tagged HP1286 was purified from the soluble fraction by chromatography on Talon resin (Clontech). To remove imidazole, the purified protein was dialyzed overnight followed by another purification step using an endotoxin removal column (Pierce). Amount of endotoxin in the purified protein was assessed by an endotoxin detection kit (Pierce) and the purified protein was concentrated using Amicon Ultra-4 centrifugal filter (Millipore). After staining with the Coomasie brilliant blue dye, the proteins appeared as a single band under reducing conditions. For the generation of an irrelevant-His-tagged (Irr-His) protein, another *H. pylori* gene (HP1563) was cloned in the same vector pET28b+ at Nde1 and XhoI sites. Irr-His was overexpressed in *E. coli* BL21 (DE3) and purified from the soluble fraction. The purified protein was of expected size (24 kDa).

### Protein extraction from *H. pylori* and immunoblotting

The methods for cell lysate preparation and immunoblotting were described previously (Tavares and Pathak, 2015). A 10 μl sample of cell lysate or 30 μl of filtered culture supernatant from each strain were used for immunoblotting. Protein A affinity-purified polyclonal antibody against rHP1286 was generated by EZbiolab (USA). AhpC antibody has been described previously (Tavares and Pathak, 2015). The band intensities of the immunoblots were quantified using the ImageJ software (NIH, Bethesda, MA, USA).

### Binding assay for rHP1286 using FACS and immunoblotting

Cells were treated with rHP1286 (1 μg/ml) for 15 min. Unbound protein was removed by washing with PBS. Cells were further incubated with an anti-His-tag antibody (Thermo fisher) (1:1,000 dilution) in FACS buffer (2% BSA in PBS) for 1 h on ice. After incubation, cells were washed twice with FACS buffer and stained with an Alexa 488-conjugated anti-mouse IgG antibody (Molecular Probes) (1:5,000 dilution) for 30 min on ice, followed by washing with FACS buffer. Binding of HP1286 was analyzed by flow cytometry using an LSRFortessa flow cytometer. FlowJo software (Tree Star, USA) was used for the data analysis. Cell lysates from untreated or rHP1286-treated samples were immunoblotted with anti-His tag antibody. Anti-actin antibody (Thermo Scientific) (1:5,000 dilution) was used to confirm equal loading.

### Binding assay for rHP1286 by immunofluorescence

RAW 264.7 cells were grown in 8-well chamber slides (Nunc) to ~50% confluence. The cells were treated with rHP1286 (1 μg/ml) for 15 min. Unbound protein was removed by washing with PBS. Cells were fixed in 2% formaldehyde for 10–15 min at room temperature, washed with PBS and were incubated in blocking buffer (2% BSA, 0.1% Tween20 in PBS) for 60 min at room temperature, followed by staining with anti-His-tag antibody (Thermo fisher) (1:500 dilution), for 1 h at room temperature. After washing with blocking buffer, cells were stained with Alexa Fluor 488-labeled secondary antibody (Molecular Probes) (1:5,000 dilution) for 1 h at room temperature. Slides were mounted with ProLong® Gold mounting medium with DAPI (4′, 6′-diamidino-2-phenylidole) (Molecular Probes). Images were captured with a fluorescent microscope (Axiovision Cell Observer HS, Carl Zeiss).

### Measurement of apoptosis

Measurement of apoptosis was performed as described previously (Pathak et al., 2013). Briefly, 1 × 10^5^ cells were stained with Annexin V-FITC antibody and Propidium Iodide (BD Biosciences) for 15 min in Annexin binding buffer (10 mM HEPES pH 7.4, 140 mM NaCl and 2.5 mM CaCl_2_) following manufacturer's instructions. Stained cells were analyzed by flow cytometry. Cells positive for Annexin V and/or PI were determined. FlowJo software (Tree Star, USA) was used for the data analysis.

### Activation assay for caspase 3/7

Caspase-3 activity was measured using the Caspase 3/7 activity assay kit (AAT Bioquest, Inc., USA) following the manufacturer's instructions. Briefly, 5 x 10^4^ cells were challenged with rHP1286 (1 μg/ml) or *E. coli* LPS (500 ng/ml) for 24 h followed by the addition of 100 μl/well of caspase 3 assay solution. The fluorescence intensity was measured (Ex/Em = 350/450 nm) after 1 h incubation. POLARstar Omega Instrument (BMG Labtech) was used for the data acquisition.

### ELISA for TNF and IL8

The amount of TNF or IL8 in the conditioned medium was measured using commercially available ELISA kit (Immunotools) following the manufacturer's instructions.

### Growth curve and viability of *H. pylori* 26695 Wt and 26695Δ*hp1286* mutant strain

The growth of *H. pylori* 26695 Wt and 26695Δ*hp1286* mutant strain in Brucella broth medium containing 8% serum was followed for 48 h by measuring the OD_600_ at specific time points. The initial optical density of each bacterial strain was adjusted to 0.05 in the referred media. *H. pylori* 26695Δ*hp1286* mutant was grown in the presence of 20 μg/ml of Chloramphenicol. To determine the viability, samples of each bacterial culture were collected at the time points of 8, 24, and 48 h, serially diluted, plated on blood agar dishes to determine the colonies forming units (CFU). Number for colonies were counted after 4–5 days of incubation under microaerophilic conditions.

### Preparation of mammalian cell lysates and immunoblotting

The method used for cell lysate preparation and immunoblotting were described previously (Pathak et al., 2013). Briefly, cell lysates were prepared by the addition of SDS-PAGE sample reducing buffer to cells after washing twice with PBS, followed by heating at 95°C for 10 min. Immunoblotting was performed using anti-phospho ERK1/2 (D13.14.4E) (1:1,000 dilution), anti-phospho-p38 (D3F9), anti-phospho-JNK (81E11), and anti-ERK1/2 (137F5) (1:1,000 dilution) antibodies from Cell Signaling Technology. Anti-phospho-c-Fos (Thr325) antibody was from Bioss antibodies. An anti-actin antibody (Thermo Scientific) (1:5,000 dilution) was used to confirm equal loading. The secondary antibodies included goat anti-rabbit IgG and goat anti-mouse IgG conjugated to IRdye800CW (Li-COR) (1:10,000 dilution). Band intensities were quantified using the ImageJ software, and actin was used to normalize the total amount of protein loaded in each well.

### Cytoplasmic and nuclear extract preparation

Extracts were prepared using a Cytoplasmic and Nuclear Extraction kit (Nordic BioSite) following the manufacturer's instructions. Briefly, cells were washed with ice cold PBS and the pellet was resuspended into the volume equal to seven times of cytoplasmic extraction reagent. After 30 min incubation on ice, the lysate was centrifuged at 16000 x g for 10 min at 4°C. Supernatant (cytoplasmic fraction) was carefully removed and the pellet was resuspended into the volume equal to two times of the nuclear extraction buffer. Lysates were placed on ice for 30 min with occasional vortexing followed by centrifugation at 16000 x g for 10 min at 4°C. Supernatant (nuclear fraction) was collected. Immunoblotting was performed using anti-phospho ERK1/2, anti-ERK1/2, anti-Histone H3 (Biolegend) and anti-α-Tubulin (B-5-1-2, Sigma) antibodies.

### Statistical analysis

All experiments were performed at least three times, each with ≤3 samples. Statistical analysis was performed using the GraphPad Prism 6.0 software. The differences between multiple groups were analyzed using one-way ANOVA. Student's *t*-test and non-parametric Man-Whitney test was used to analyze difference between two groups. Values of *p* < 0.05 were considered statistically significant.

## Results

### Expression and release of HP1286 by *H. pylori*

**Figure 1:**
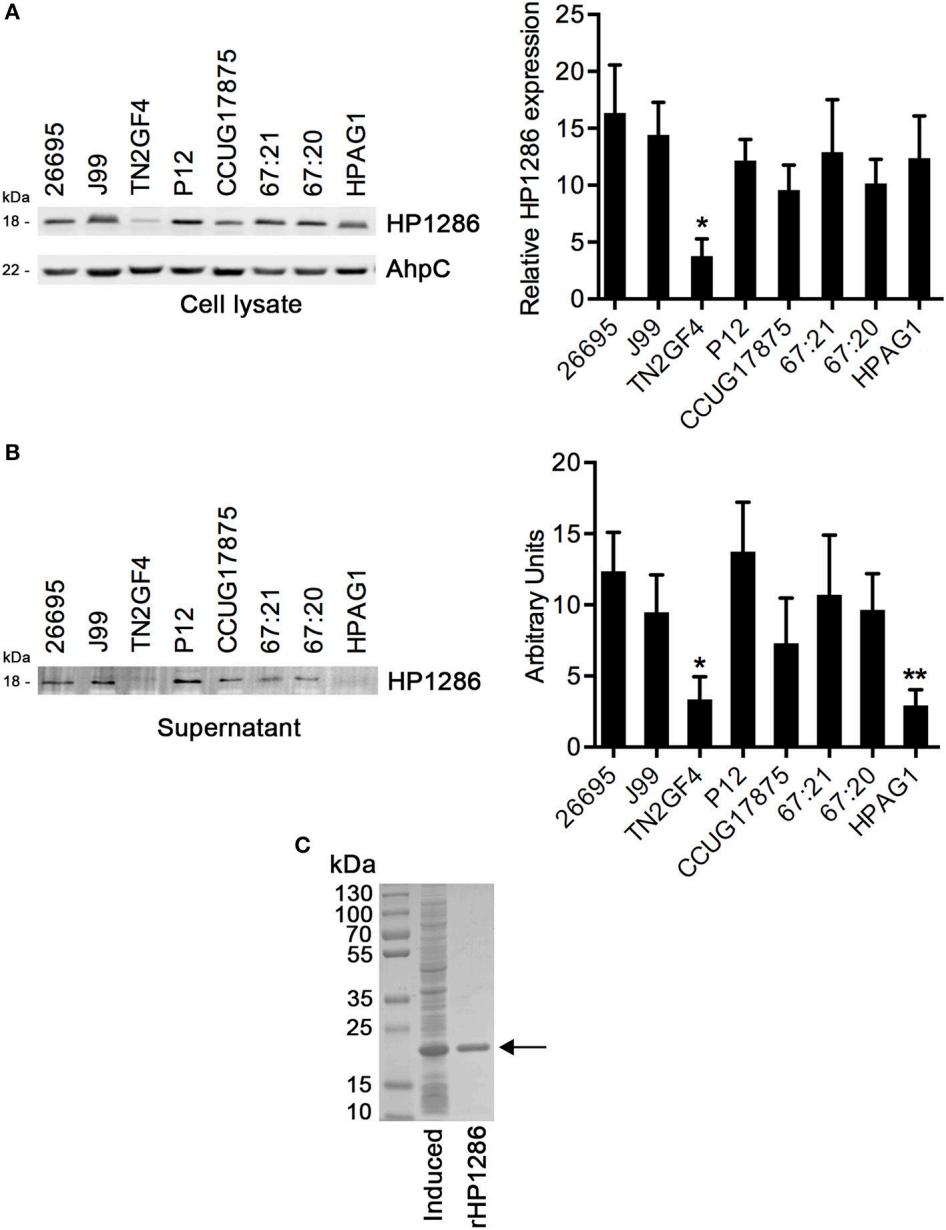
**Expression and release of HP1286 in different *H. pylori* strains**. Whole cell lysates **(A)** and cell culture supernatant **(B)** from equal number of cells of different *H. pylori* strains were immunoblotted with anti-HP1286 antibody. The blot was reprobed with anti-AhpC antibody. Data shown is the representative of six independent experiments. The bar diagram indicates the band intensities normalized to the loading control **(A)** and the mean ± *SD* of band intensities **(B)**. Statistically significant differences were observed between TN2GF4 and other strains in both whole cell lysate **(A)** and culture supernatant **(B)**, which is indicated by ^*^(*p* < 0.05). The strain HPAG1 showed statistically significant difference from all strains except TN2GF4 in the culture supernatant **(B)**, which is indicated by ^**^(*p* < 0.05) **(C)** rHP1286 was separated on 4–15% SDS-polyacrylamide gel under reducing conditions followed by staining with Coomassie blue.

HP1286 is highly conserved among *H. pylori* strains. The level of HP1286 expression among *H. pylori* strains was tested using an antibody raised against rHP1286. Although HP1286 expression was observed in all strains, the level of expression varied significantly among some strains (Figure 1A). The expression of the protein in the strain TN2GF4 was significantly lower than all other strains. The same lysates were blotted with antibodies against AhpC, a highly abundant protein of *H. pylori*. No difference in the level of AhpC expression was observed among the tested strains (Figure 1A). The presence of a signal peptide (1–17 aa) at the N-terminus, suggested the secretory nature of HP1286 and the release of protein in the external medium has been reported in previous studies with *H. pylori* strains NCTC 11637 and ATCC43504 (Kim et al., 2002; Toledo et al., 2002). We further assessed the level of protein HP1286 released in the culture medium of several other *H. pylori* strains. As observed in the cell culture lysate, there were significant differences in the level of released protein among strains (Figure 1B). Interestingly, for the strain HPAG1 the level of released protein did not correlate with the expression level in the cell culture lysates. As shown in Figures 1A,B, although the level of expression of HP1286 in HPAG1 lysate was higher or similar to all other strains except TN2GF4, the released level of HP1286 protein in the external medium of the strain HPAG1 was significantly lower than all strains except TN2GF4.

In order to study the effect of HP1286 on host cells, a sequence encoding amino acids 18 to 182 of HP1286 from *H. pylori* strain 26695 was cloned, overexpressed and purified as a His-tag protein from the soluble fraction of *E. coli*. N-terminal amino acids 1–17 were the predicted signal peptide. Hence, it was excluded from the HP1286 construct. The recombinant purified HP1286 (rHP1286) had the expected size (Figure 1C).

### rHP1286 binds to macrophages

**Figure 2:**
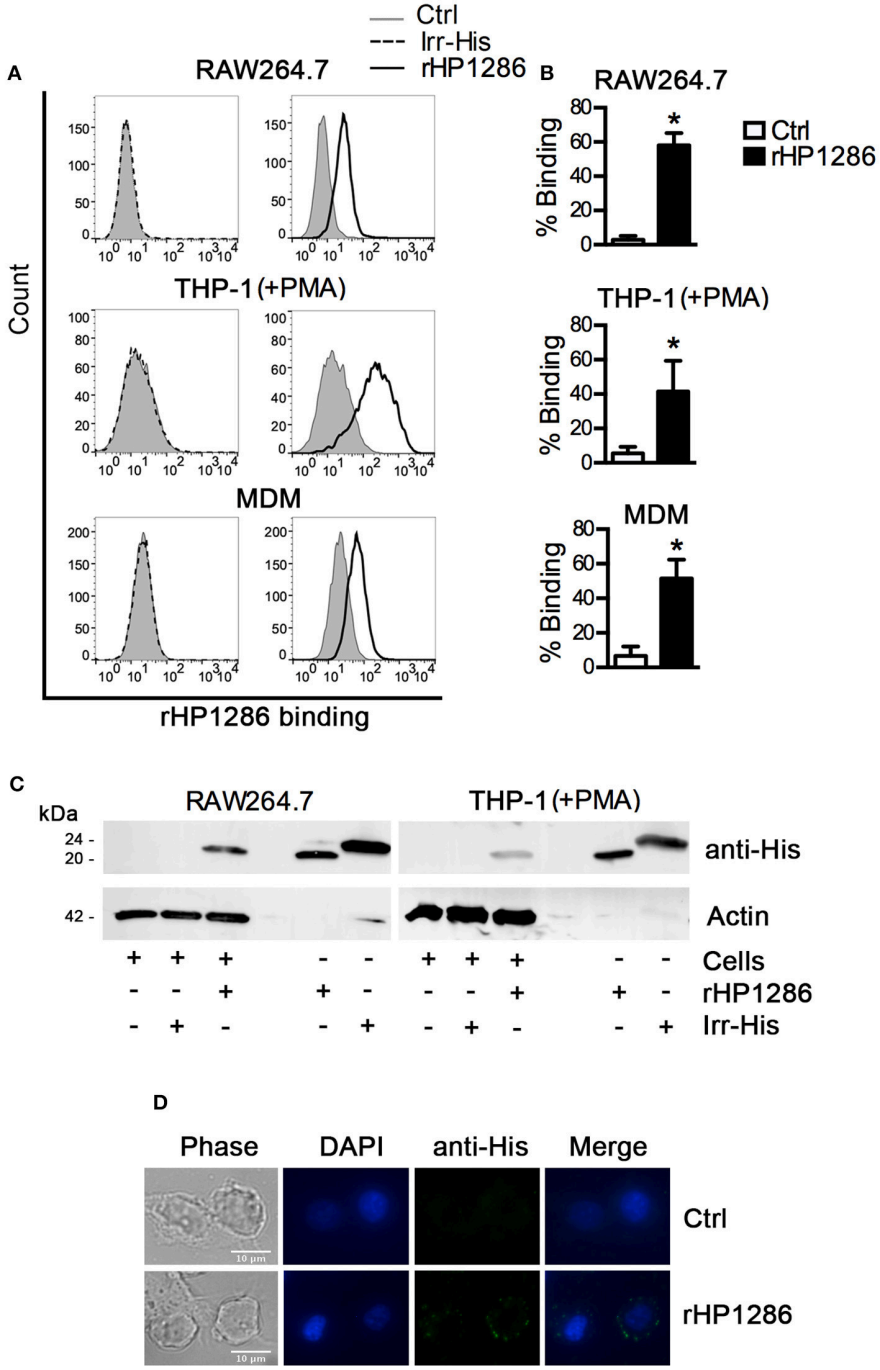
**rHP1286 binds to macrophages. (A)** Cells were incubated either with buffer (Ctrl) or with rHP1286 (1 μg/ml) or an irrelevant His-tagged protein (Irr-His, 1 μg/ml) for 15 min, followed by washing with PBS to remove unbound protein and subsequent staining with anti-His-tag antibody and Alexa Fluor 488 conjugated secondary antibody. Binding was analyzed by flow cytometry. One representative data from results obtained in ≥3 independent experiments is shown. **(B)** The results obtained in the binding assays are depicted as percentage binding of rHP1286 to cells in RAW264.7 (five experiments), THP-1 (+PMA) (three experiments) and MDM (three donors). Values indicate mean ± SD. Statistically significant differences are indicated by ^*^(*p* < 0.05). **(C)** RAW264.7 and THP-1 (+PMA) cells were incubated with rHP1286 or Irr-His as described above followed by the preparation of cell lysates in SDS-PAGE sample buffer. Lysates were immunoblotted with anti-His-tag antibody and the blots were reprobed with anti-actin antibody. One representative data from results obtained in ≥3 independent experiments is shown. **(D)** RAW264.7 cells were incubated with rHP1286 as described above. Immunofluorescence analysis was performed after staining with anti-His-tag antibody and Alexa Fluor 488-labeled secondary antibody. Cells were mounted in ProLong® Gold mounting medium with DAPI. Scale bar, 10 μm. One representative experiment of four is shown.

Considering the release of HP1286 in the culture supernatant of different strains of *H. pylori*, we further accessed the binding of rHP1286 to macrophages. Binding of rHP1286 was studied using several methods. Macrophages were incubated with various concentrations of rHP1286, followed by washing of unbound protein and staining with anti-His-tag antibody and Alexa 488-conjugated secondary antibody. FACS analysis indicated the binding of rHP1286 to human monocytic cell line THP-1 (41.6 ± 6.7%) differentiated into macrophages like cells by PMA treatment, RAW264.7 (58.02 ± 3.02%) and human monocyte-derived macrophages (MDM) (51.6 ± 4.4%) (Figures 2A,B). An irrelevant His-tagged protein was used to rule out the possibility of the His-tag contributing to the binding of purified protein to cells. Binding of the irr-His to macrophages was not detected under similar conditions (Figure 2A). The interaction of rHP1286 with macrophages was also detected by immunoblotting of rHP1286-treated cell lysates with anti-His-tag antibody (Figure 2C). Immunofluorescence staining further confirmed the binding of rHP1286 to macrophages (Figure 2D). Taken together, these results indicated that rHP1286 interacts with macrophages.

### rHP1286 induces apoptosis in macrophages

**Figure 3:**
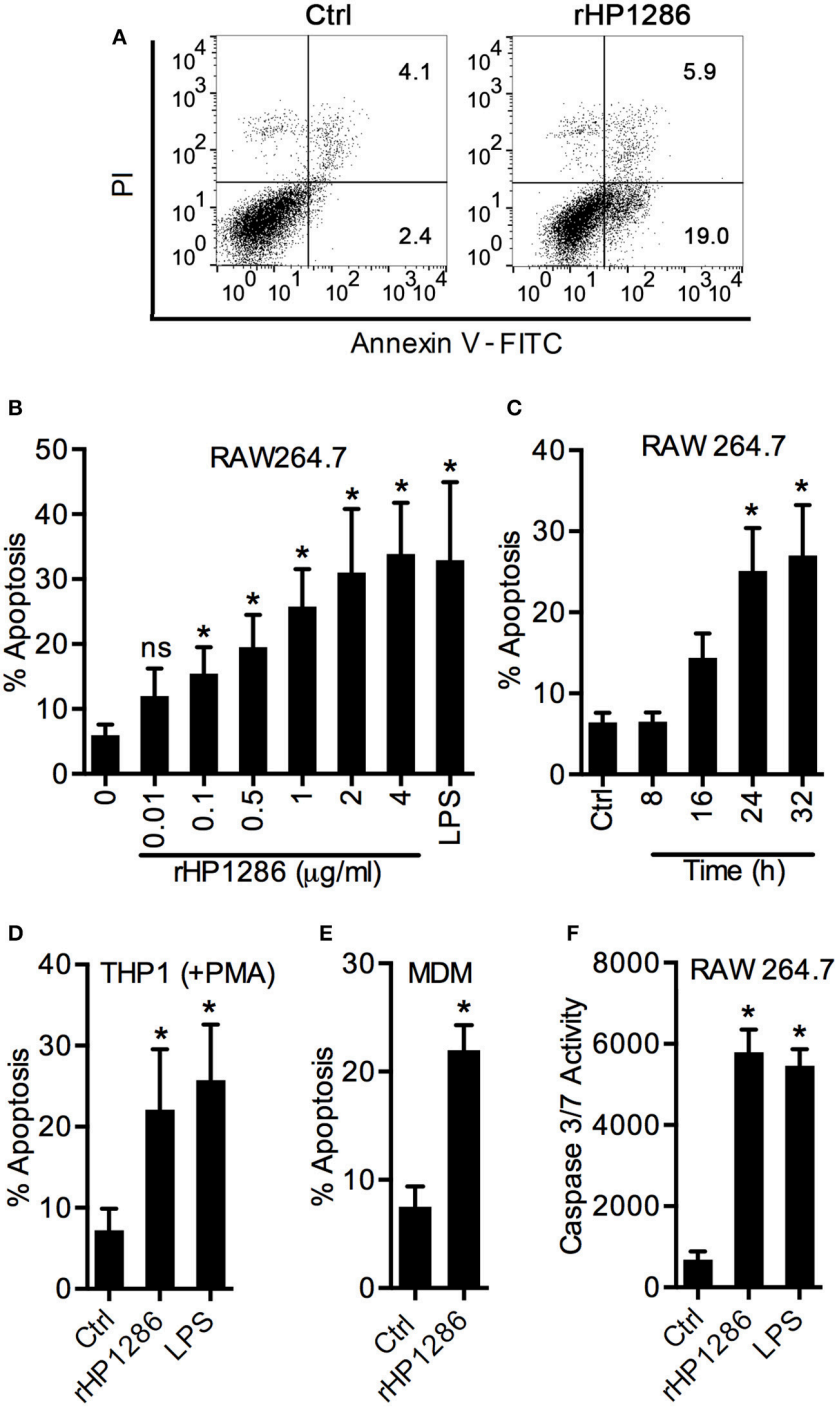
**rHP1286 induces apoptosis in macrophages. (A)** RAW 264.7 cells were incubated either with protein storage buffer (Ctrl) or rHP1286 (1 μg/ml) for 24 h. Cells were washed, stained with Annexin V-FITC antibody and PI as described in material and methods and analyzed by flow cytometry. Result shown is the representative of results obtained in more than four independent experiments performed with at least duplicate samples. RAW264.7 cells were treated with various concentrations of rHP1286 as indicated in figure legends or *E. coli* LPS (500 ng/ml) for 24 h **(B)** or treated with rHP1286 (1 μg/ml) for different time periods **(C)**. Apoptotic cells were analyzed by flow cytometry. Values indicate mean ± SD of four independent experiments. ns indicates statistically non-significant. PMA-treated THP-1 **(D)** and MDM **(E)** cells were incubated either with rHP1286 (1 μg/ml) or *E. coli* LPS (500 ng/ml) for 24 h. Apoptotic cells were analyzed by flow cytometry after staining with Annexin V-FITC and PI. Values in panel D indicates mean ± SD of five independent experiments. For MDM, values indicate mean ± *SD* of apoptosis in three donors. **(F)** RAW264.7 cells were treated either with buffer (Ctrl) or rHP1286 (1 μg/ml) or *E. coli* LPS (500 ng/ml) for 24 h. Caspase 3/7 activity was measured. Data shown is mean ± *SD* of four independent experiments performed in triplicates. Statistically significant differences are indicated by ^*^(*p* < 0.05).

Considering the binding of rHP1286 to macrophages, we were further interested in studying the host cell responses in rHP1286-challenged macrophages. We observed morphological changes associated with apoptosis in rHP1286-challenged macrophages. To confirm an increase in apoptosis, RAW264.7 cells were treated with various concentrations of rHP1286 for 8–32 h followed by staining with Annexin V-FITC and PI. A representative FACS analysis of apoptosis is shown in Figure 3A. Apoptosis was induced in a dose- and time-dependent manner (Figures 3B,C). In addition to RAW264.7 cells, rHP1286 also induced apoptosis in PMA treated-THP-1 cells (Figure 3D), and MDM (Figure 3E). Activation of caspases is an important indicator of apoptotic cell death. Significantly higher caspase 3/7 activity was observed in rHP1286-challenged RAW264.7 cells (Figure 3F). Taken together, these results indicated that rHP1286 induces apoptosis in macrophages.

**Figure 4:**
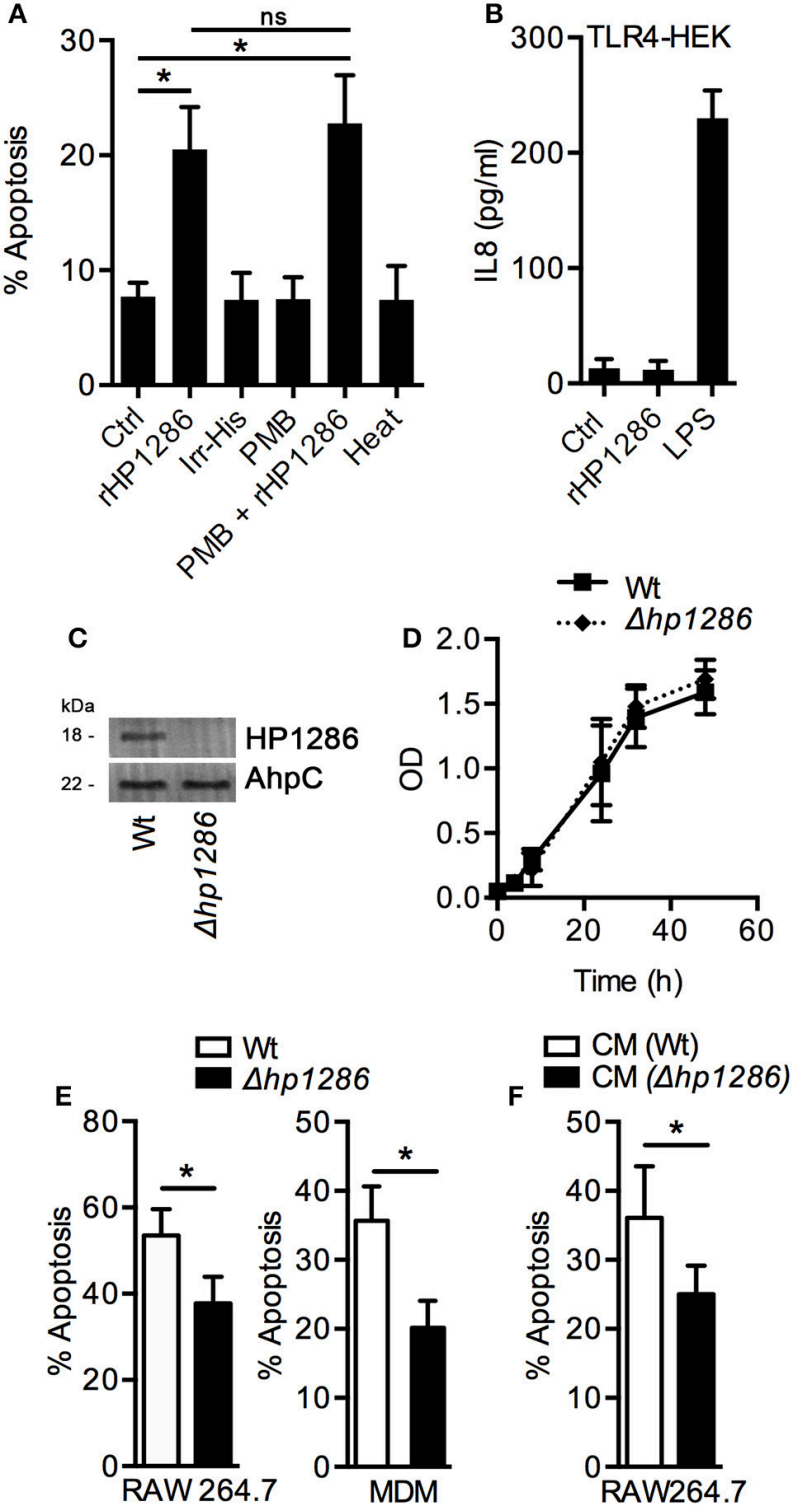
**Apoptosis-inducing ability is a property specific to HP1286. (A)** RAW264.7 cells were treated for 24 h with buffer (Ctrl) or rHP1286 (1 μg/ml) or an irrelevant His-tagged protein (Irr-His, 1 μg/ml). In another set of experiments, rHP1286 was treated with polymyxin B (PMB + rHP1286) for 1 h or rHP1286 was subjected to boiling for 30 min (heat) before treatment of cells. Apoptotic cells were analyzed by flow cytometry after staining with Annexin V-FITC and PI. Values indicate mean ± *SD* of four independent experiments. Statistically significant differences are indicated by ^*^(*p* < 0.05). ns indicates statistically non-significant. **(B)** TLR4-HEK cells were treated with rHP1286 (1 μg/ml) or *E. coli* LPS (500 ng/ml) for 16 h. IL-8 level in the cell culture supernatant was determined by ELISA. Data shown is the mean ± *SD* of two independent experiments. **(C)** Cell lysates from *H. pylori* 26695 wild-type strain (Wt) or the mutant strain (Δ*hp1286*) were immunoblotted with anti-HP1286 antibody. Blots were reprobed with anti-AhpC antibody to confirm equal loading. Data shown is representative of results obtained in three independent experiments. **(D)** *H. pylori* 26695 Wt or Δ*hp1286* were grown in liquid culture for 48 h and optical density was measured at indicated time points. Data shown is the results obtained from three independent experiments. **(E)** RAW264.7 and MDM cells were infected either with *H. pylori* 26695 Wt or Δ*hp1286* at MOI100. Apoptotic cells were analyzed by flow cytometry. Data shown is the results obtained from three independent experiments (RAW264.7) or three donors (MDM). **(F)** RAW264.7 cells were incubated in the conditioned medium (CM) of *H. pylori* 26695 Wt or Δ*hp1286* for 24 h. Apoptotic cells were analyzed by flow cytometry. The error bars represent standard deviations. Statistically significant differences are indicated by ^*^(p < 0.05).

Various control experiments were performed to confirm the specific effect of rHP1286 on macrophages. An irrelevant His-tag protein was used in parallel to rule out the possibility of the His-tag contributing to the observed effects. Under similar conditions, Irr-His failed to induce apoptosis in macrophages (Figure 4A). LPS antagonist PMB had no effect on the apoptosis-inducing ability of rHP1286 (Figure 4A). Furthermore, rHP1286 was incubated at 95°C for 10 min before treatment of the macrophages. The heat treatment resulted in the loss of apoptosis-inducing ability, indicating that the effect is due to the HP1286 protein (Figure 4A). In addition to these controls, experiments were also performed using HEK cells, which are naturally devoid of receptors for LPS signaling, as well as HEK-TLR4 cells that were stably transfected with LPS signaling receptors TLR4/MD2/CD14. rHP1286 failed to induce IL8 in HEK-TLR4 cells at concentrations used in this study (Figure 4B). As expected, our positive control *E. coli* LPS induced a significantly higher amount of IL8 in HEK-TLR4 cells (Figure 4B). Collectively, these results ruled out the possibility of LPS contamination in rHP1286.

An isogenic mutant strain *H. pylori* 26695 was constructed to confirm the apoptosis-inducing ability of endogenous HP1286, The *hp1286* gene was disrupted by insertion of a chloramphenicol cassette and lack of HP1286 protein expression was verified by immunoblotting with an antibody-raised against rHP1286 (Figure 4C). First, we questioned whether the protein could have an effect on bacterial growth and/or viability. Growth and viability of *H. pylori* 26695 Wt and 26695Δ*hp1286* was monitored up to 48 h. No significant differences in growth were seen between the Wt and the mutant strain (Figure 4D), which ruled out the possibility of the involvement of HP1286 in the bacterial growth. In comparison to the 26695 Wt strain, significantly reduced apoptosis was observed in RAW264.7 and MDM infected with 26695Δ*hp1286* confirming that the endogenous HP1286 regulates macrophage apoptosis (Figure 4E). Furthermore, significantly less apoptosis was observed in RAW264.7 cells incubated in culture supernatant (CM) from 26695Δ*hp1286* (Figure 4F).

### The effect of HP1286 on various cell types

**Figure 5:**
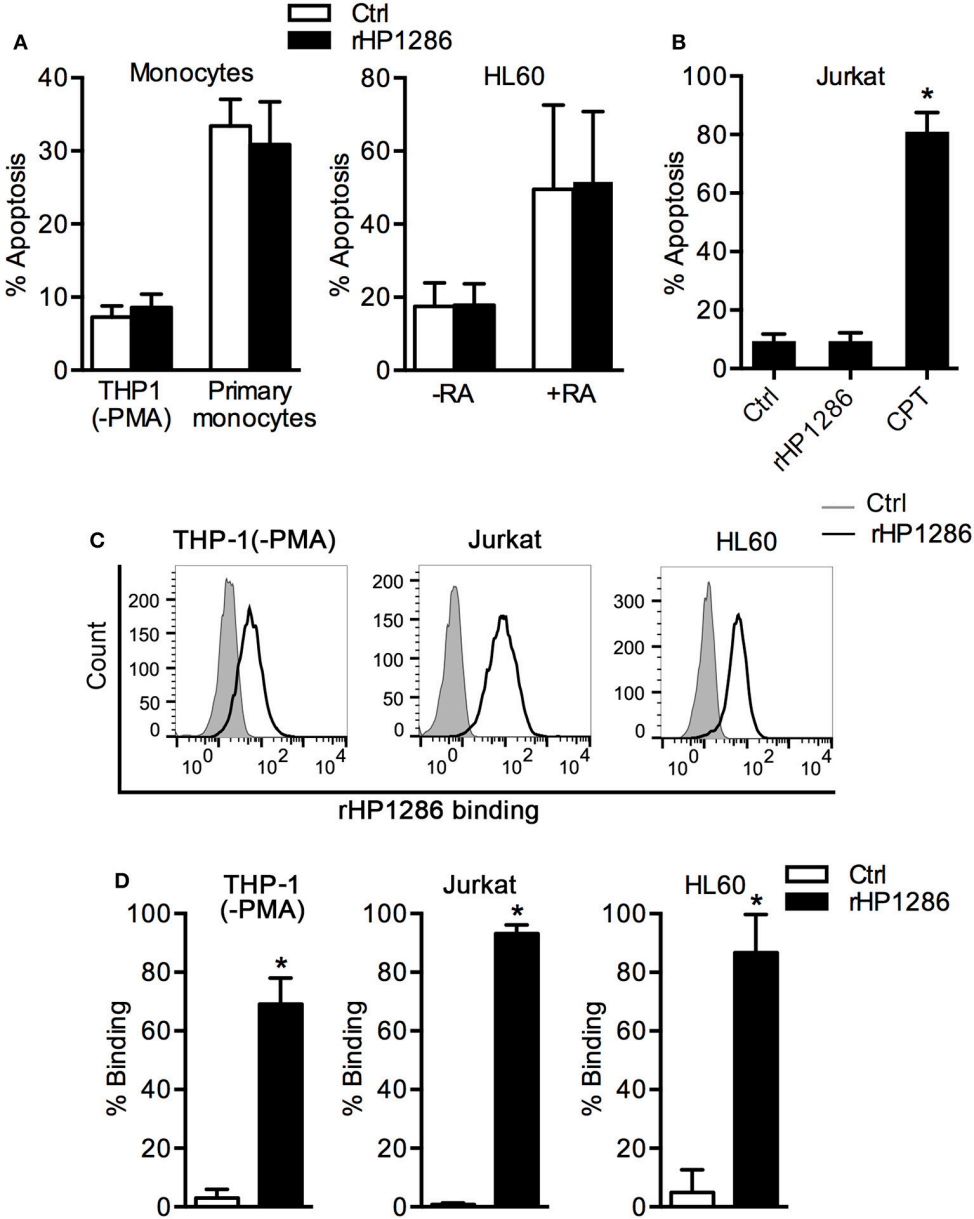
**The effect of rHP1286 on various cell types. (A)** THP-1 (−PMA) cells, primary human monocytes and untreated (−RA) or retinoic acid treated (+RA) HL60 cells were incubated with buffer (Ctrl) or rHP1286 (1 μg/ml) for 24 h. **(B)** Jurkat cells were treated for 24 h with buffer (Ctrl) or rHP1286 (1 μg/ml) or Camptothecin (CPT, 10 μM). Cells were stained with Annexin V-FITC and PI and the percentage of apoptotic cells were analyzed by flow cytometry. Values indicate mean ± *SD* of four independent experiments (THP-1, HL60 and Jurkat) and three donors (primary monocytes). Statistically significant differences are indicated by ^*^(*p* < 0.05). **(C)** Different cells as indicated in the figure legends were incubated either with protein storage buffer (Ctrl) or with rHP1286 (1 μg/ml) for 15 min. Cells were washed, stained with anti-His-tag antibody and Alexa Fluor 488-conjugated secondary antibody followed by flow cytometry analysis. Result shown is the representative of results obtained in ≥3 independent experiments performed with at least duplicate samples. **(D)** The results obtained in the binding assays are depicted as percentage binding of rHP1286 to cells. Values indicate mean ± *SD*. Statistically significant differences are indicated by ^*^(*p* < 0.05).

It has been reported that rHP1286 induces apoptosis in gastric epithelial cells (Li et al., 2012), and we observed apoptosis in HP1286-challenged macrophages. We further studied the apoptosis-inducing ability of rHP1286 on other possible target cells types such as monocytes, T cells, and neutrophils. Under similar conditions, rHP1286 failed to induce apoptosis in undifferentiated THP-1 cells (human monocytic cell line) and human primary monocytes (Figure 5A). HL-60 (human promyelocytic leukemia) cells were either undifferentiated or differentiated to a neutrophil-like phenotype. High level of background apoptosis was observed in the retinoic acid-differentiated HL-60 cells, which is in agreement with previous observations (Marshall et al., 2008; Sjölinder et al., 2012). The rate of apoptosis was not affected in rHP1286-challenged undifferentiated or differentiated HL-60 cells (Figure 5A). Similarly, rHP1286 did not induce significantly higher apoptosis in Jurkat cells (human T lymphocyte cells), whereas our positive control Camptothecin (CPT) did induce apoptosis (Figure 5B). The binding of rHP1286 was observed in undifferentiated THP-1 (69.5 ± 4.5%), Jurkat (93.1 ± 2.02%), and HL60 (86.7 ± 5.31%) (Figures 5C,D). Taken together, these results indicated that although rHP1286 interacts with various cells, it induces apoptosis in only certain cell types.

### rHP1286-induced macrophage apoptosis is independent of TNF

**Figure 6:**
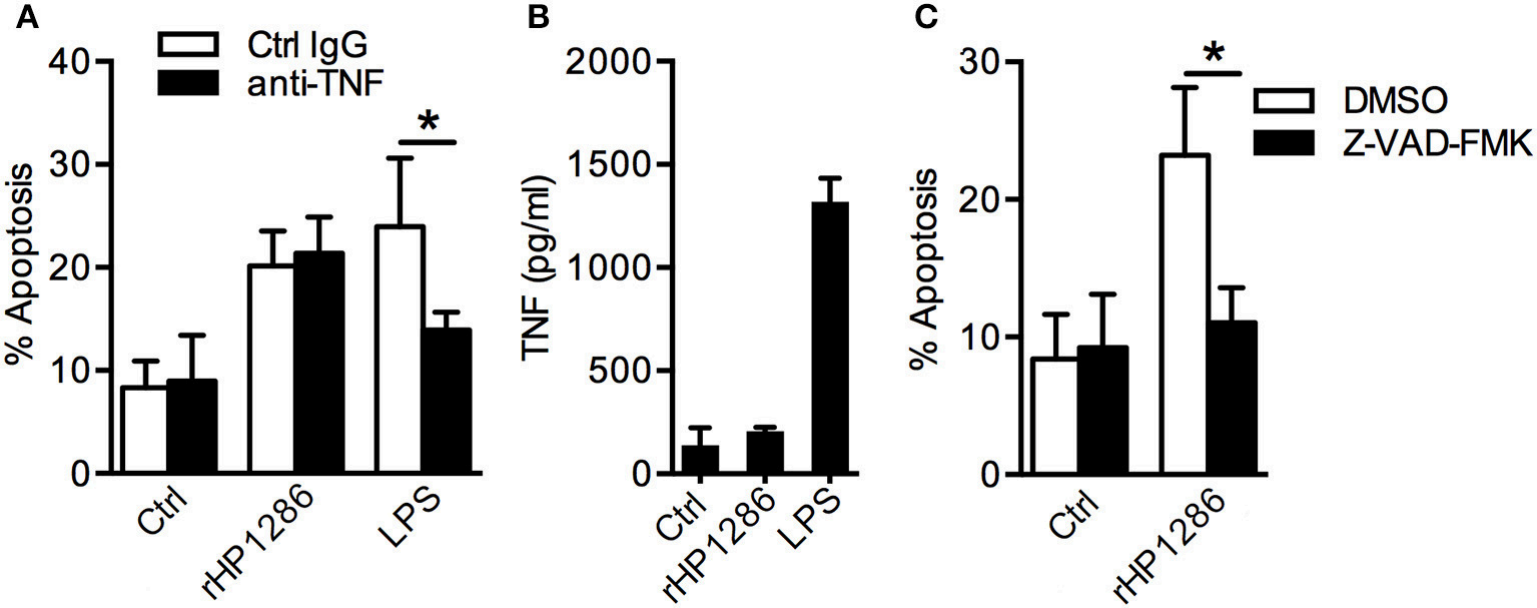
**The role of TNF and caspases in rHP1286-induced macrophage apoptosis. (A)** RAW264.7 cells were pretreated either with isotype control IgG or TNF neutralizing antibody (10 μg/ml) for 1 h followed by treatment with either rHP1286 (1 μg/ml) or *E. coli* LPS (500 ng/ml) for 24 h. Cells were stained with Annexin V-FITC and PI and the percentage of apoptotic cells were analyzed by flow cytometry. Values indicate mean ± *SD* of four independent experiments. **(B)** RAW264.7 cells were treated either with buffer (Ctrl) or rHP1286 (1 μg/ml) or *E. coli* LPS (500 ng/ml) for 16 h. The release of TNF in the cell culture supernatant was measured by ELISA. The data shown is the mean ± *SD* of four independent experiments. **(C)** Percentage of apoptotic cells was determined in RAW264.7 cells treated either with the vehicle control (DMSO) or pan-caspase inhibitor Z-VAD-FMK (20 μM) for 1 h followed by 24 h incubation with rHP1286 (1 μg/ml). The data shown is the mean ± *SD* of four independent experiments. Statistically significant differences are indicated by ^*^(*p* < 0.05).

*H. pylori* induces TNF in macrophages and TNF has been shown to regulate macrophage apoptosis (Rizwan et al., 2008; Alvi et al., 2011; Pathak et al., 2013). We therefore studied the possibility of TNF-dependent apoptosis induction in rHP1286-challenged macrophages. Pretreatment of macrophages with anti-TNF neutralizing antibody had no effect on rHP1286-induced apoptosis, whereas LPS-induced apoptosis was significantly blocked (Figure 6A). Furthermore, rHP1286 failed to induce significantly higher level of TNF in the culture medium of macrophages up to 16 h after treatment at the concentrations that induced apoptosis in macrophages (Figure 6B). Significantly higher level of TNF was detected in the culture supernatant of LPS-treated macrophages (Figure 6B). Collectively, these results indicated that HP1286-induced macrophage apoptosis does not depend on TNF induction. In addition, rHP1286-induced macrophage apoptosis was significantly inhibited by pan-caspase inhibitor, suggesting that rHP1286 triggered caspase-dependent macrophage apoptosis (Figure 6C).

### rHP1286-induced macrophage apoptosis is dependent on ERK MAPK

**Figure 7:**
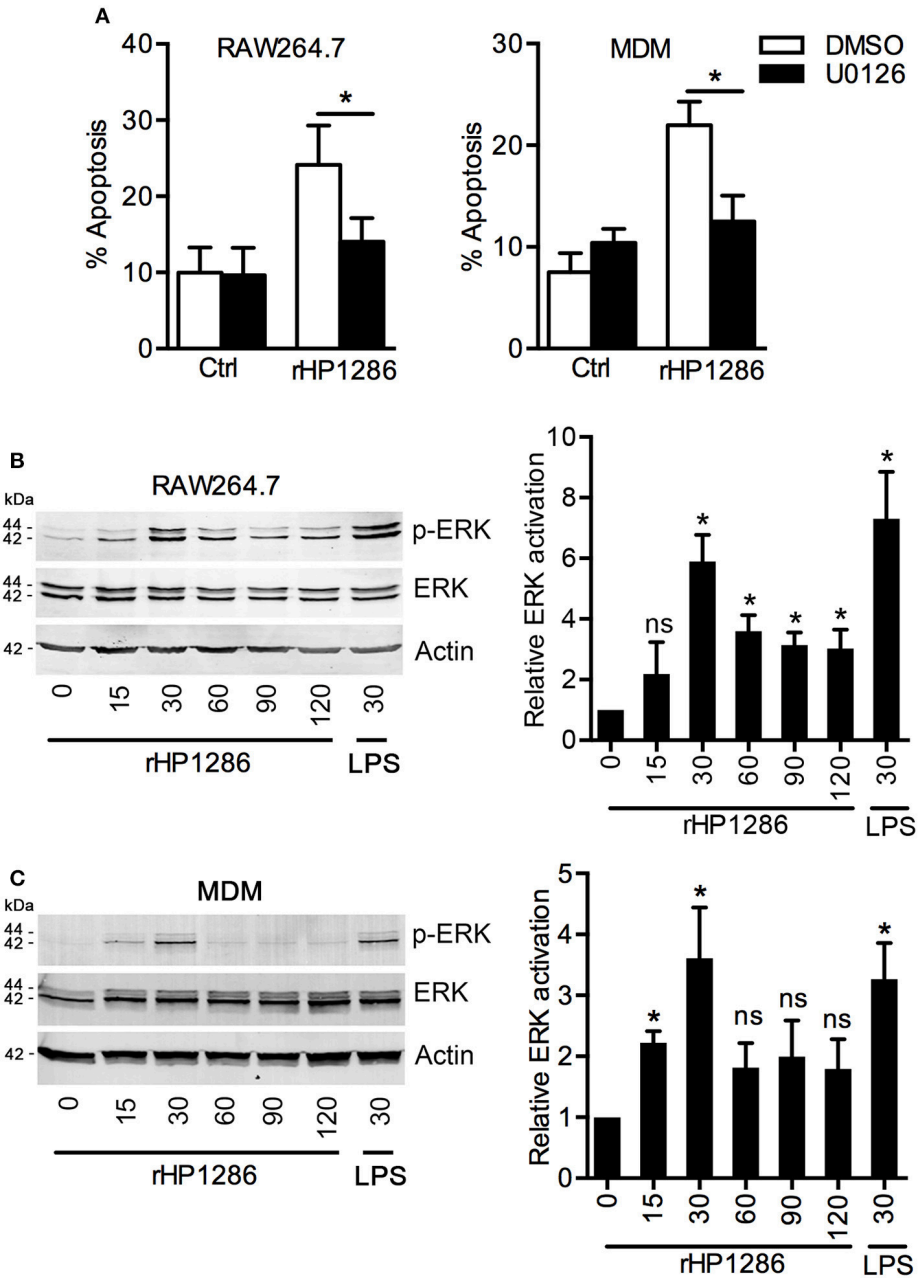
**The role of ERK MAPK in rHP1286-induced macrophage apoptosis. (A)** RAW264.7 and MDM cells were pretreated either with vehicle control (DMSO) or U0126 (10 μM) for 30 min followed by treatment with rHP1286 (1 μg/ml). Apoptotic cells were analyzed by flow cytometry. The data shown is the mean ± *SD* of three independent experiments (RAW264.7) or three donors (MDM). Statistically significant differences are indicated by ^*^(*p* < 0.05). RAW264.7 cells **(B)** or MDM **(C)** were treated with rHP1286 (1 μg/ml) for indicated time periods. As a positive control for ERK activation, RAW264.7 cells were treated with *E. coli* LPS for 30 min. Western blotting was performed with anti-phospho-ERK antibody. Blots were reprobed with anti-ERK and anti-actin antibody to ensure equal loading. Data shown is the representative of four independent experiments (RAW264.7) and three donors (MDM). The bar diagram indicates band intensities in western blots normalized to the actin control. ^*^(*p* < 0.05) indicates statistically significant differences.

ERK MAPK has been reported to regulate apoptosis in *H. pylori* infected macrophages (Asim et al., 2010). We therefore, studied the possible involvement of ERK MAPK in rHP1286-induced macrophage apoptosis. Macrophages were pre-treated with U0126 to block ERK MAPK followed by treatment with rHP1286. Blocking of ERK MAPK resulted in significantly lower apoptosis in rHP1286-treated RAW264.7 and MDM cells (Figure 7A). Similar results were obtained in THP-1 (+PMA) cells (data not shown). Further, to confirm the activation of ERK MAPK in rHP1286-challenged macrophages, the level of phosphorylated ERK 1/2 was assessed. rHP1286 was found to induce ERK activation in a time-dependent manner in both cell types (Figures 7B,C). The ERK activation kinetics differed slightly between the two cell types. Significantly higher ERK MAPK activation in MDM was observed as early as 15 min, whereas maximum ERK activation was observed for both cell types after 30 min of rHP1286 treatment. A densitometry analysis of ERK phosphorylation kinetics from ≥3 independent experiments is shown in Figures 7B,C.

**Figure 8:**
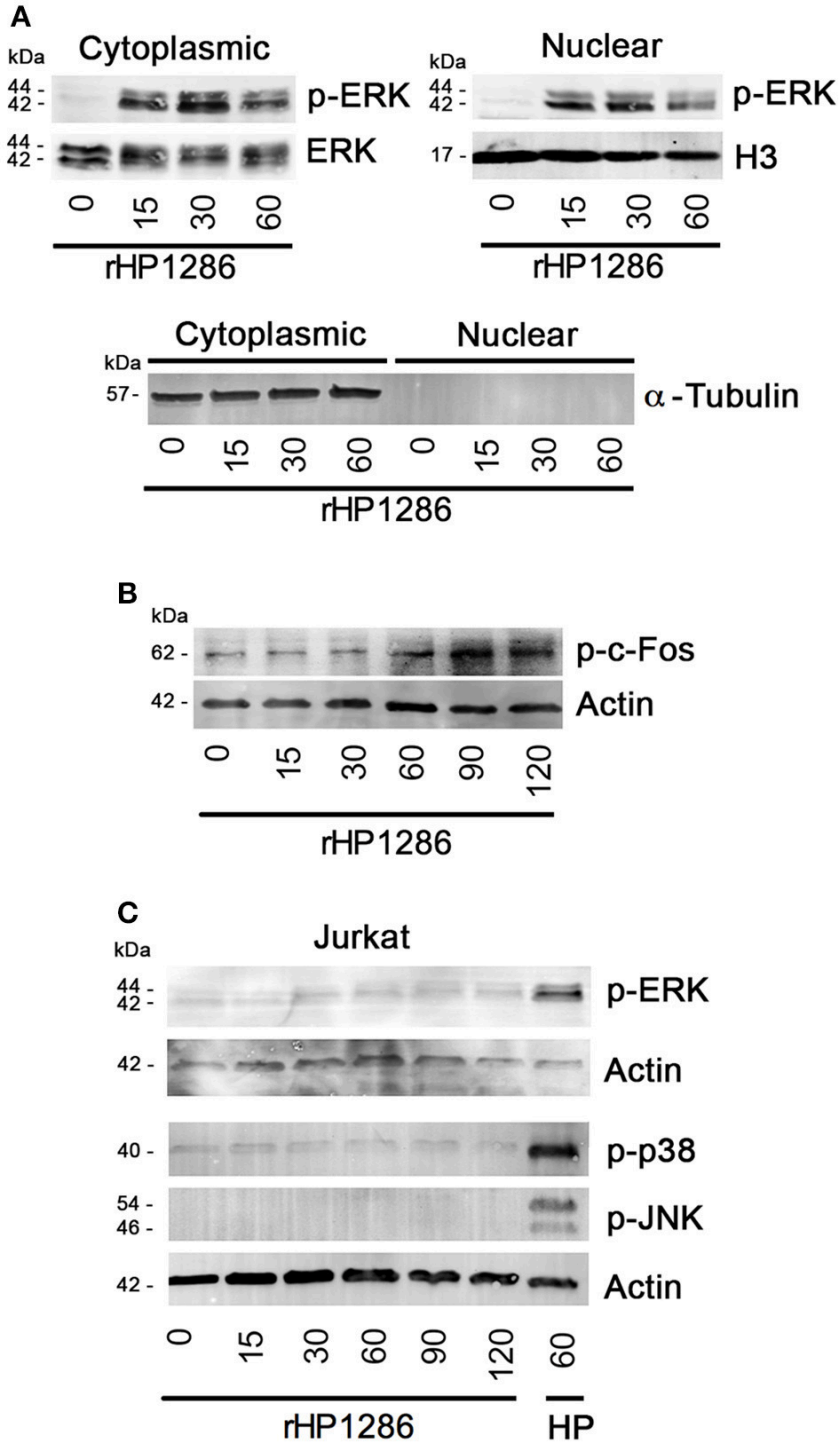
**The effect of rHP1286 on MAPKs and c-Fos activation in different cell types. (A)** Cytopalsmic and nuclear fraction of rHP1286-treated cells were immunoblotted with anti-phospho-ERK antibody. Blots were reprobed with anti-ERK or Histone H3 antibody to conform equal loading. Cytoplasmic and nuclear fractions were immunoblotted with α-Tubulin antibody to check purity. Data shown is the representative of four independent experiments. **(B)** RAW264.7 cells were treated with rHP1286 (1 μg/ml) for indicated time periods. Cell lysates were immunoblotted with anti-phosho-c-Fos (Thr325) antibody and reprobed with anti-actin antibody to confirm equal loading. Data shown is the representative of four independent experiments. **(C)** Jurkat cells were treated with rHP1286 (1 μg/ml) for indicated time periods. Cell lysates were immunoblotted with anti-phospho-ERK, anti-phospho-p38 and anti-phospho-JNK antibodies. As a positive control, Jurkat cells were infected with *H. pylori* 26695 (HP) for 60 min. Blots were reprobed with anti-actin antibody to confirm equal loading. Data shown is the representative of three independent experiments.

It has been reported previously that ERK-dependent apoptosis requires nuclear translocation of phosphorylated ERK (Asim et al., 2010; Teixeiro and Daniels, 2010); therefore we further studied the level of phosphorylated ERK in cytoplasmic and nuclear fractions of macrophages. Phosphorylated-ERK was detected in both cytoplasmic and nuclear fractions of rHP1286-treated RAW264.7 cells (Figure 8A). Furthermore, rHP1286 was found to induce phosphorylation of c-Fos at Thr^325^ in a time-dependent manner in RAW264.7 cells (Figure 8B).

Depending on the stimulus used, MAPKs have been shown to differentially regulate apoptosis in different cell types (Wada and Penninger, 2004). Considering the binding of rHP1286 to other cell types but absence of apoptosis induction, we were further interested in identifying the differences in MAPK activation. Under similar conditions, rHP1286 failed to activate ERK, p38 and JNK MAPKs in Jurkat cells (Figure 8C).

## Discussion

Macrophages play an essential role in the host responses against *H. pylori*-derived factors and also from signals from epithelial cells. In order to establish a persistent infection, *H. pylori* must modulate the effector functions of the macrophages for their own benefit and survival. Although *H. pylori* has been demonstrated to modulate the macrophage responses in various studies (Gobert et al., 2002a; Chaturvedi et al., 2004; Asim et al., 2010), only a small number of macrophage function-regulating *H. pylori* virulence factors have been identified to date, such as HP0940, HP0986, Urease, JHP0290 (Harris et al., 1998; Alvi et al., 2011; Pathak et al., 2013; Tenguria et al., 2014). In this study, we have identified HP1286 as a novel regulator of macrophage apoptosis. The identification and characterization of unknown or less-studied virulence factors is very important when considering the fact that we still do not know fully how or which factors actually determine the disease development in a small percentage of infected individuals.

Sequence homology analysis among *H. pylori* strains indicated that HP1286 is highly conserved (Li et al., 2012). We observed significant differences in the level of expression of the protein among some *H. pylori* strains. Furthermore, the amount of released protein in the culture supernatant also varied significantly among some strains. These differences could be an intrinsic property of the isolate or may be due to the differences in the encountered environmental conditions at the sites of origin of the isolates, such as pH, nutrients, level of mucins, etc. *H. pylori* shows high genetic diversity and it is believed that the final disease development at least partially depends on strain-specific features. In several independent studies, differences in the level of expression or release of *H. pylori* proteins have been observed (Hennig et al., 2004; Suganuma et al., 2005; Devi et al., 2014; Tavares and Pathak, 2015). These differences in the level of expression or release could potentially determine the virulence of different strains. Further studies are required to understand the mechanisms that regulate the expression and release of HP1286 and the precise relationship between expression level and the virulence of the bacterial strains.

*H. pylori* infection induces a chronic (lymphocytic) and active (neutrophilic) inflammatory responses. Although *H. pylori* is generally considered as a non-invasive pathogen, it can disrupt the gastric epithelial cell layer, and the presence of bacterial proteins in the lamina propria has been reported (Mai et al., 1992; Semino-Mora et al., 2003). In a majority of gastritis and gastric cancer cases, *H. pylori* was found to be in direct contact with immune cells (Moese et al., 2007). A series of studies observed the responses of monocytes, neutrophils, macrophages, T-cells, and B-cells to *H. pylori* and bacterium derived-products (Boncristiano et al., 2003; Sundrud et al., 2004; Bussiere et al., 2006; D'Elios et al., 2007; Schmees et al., 2007; Asim et al., 2010). Studies have also shown that contact with live bacteria is not required and responses were observed when immune cells were separated by a filter or treated with *H. pylori* water extract or whole cell lysate (Gobert et al., 2002a,b; Chaturvedi et al., 2004). HP1286 was reported to induce apoptosis in gastric epithelial cell line AGS (Li et al., 2012). Considering the effect of HP1286 and other known epithelial cell layer disrupting factors we further studied the possible interaction of HP1286 with other target cell types. rHP1286 was found to interact with various cell types such as macrophages, monocytes, neutrophils and T-cells, indicating that the protein is recognized by certain receptor(s), which is/are present on multiple cell types. In comparison to macrophages, better binding of rHP1286 was observed in other cell types. Considering the differences in the degree of binding of the rHP1286 among cell types under similar experimental conditions, it is expected that the level of the receptor expression possibly vary among different cell-types. Currently, we are working on the identification of the host cell surface receptor that recognizes rHP1286.

Exposure to rHP1286 resulted in a dose- and time-dependent apoptosis in macrophages. However, the same protein failed to induce apoptosis in primary monocytes, T cells and neutrophil-like HL 60 cells under similar conditions. These observations suggest that although rHP1286 interacts with several cell types, it might have effects that are specific to each cell type. This is not an unexpected phenomenon especially when considering the pathogenesis of a highly successful pathogen such as *H. pylori*. Recently, we reported that another *H. pylori* secreted protein JHP0290 was able to bind to both macrophages and gastric epithelial cells. However, this interaction resulted in pro-apoptotic signaling in macrophages, whereas binding to gastric epithelial cells resulted in proliferative and anti-apoptotic signaling pathways (Pathak et al., 2013; Tavares and Pathak, 2015). Differences in the activation of the host cell pro-apoptotic and anti-apoptotic signaling pathways in different cell types could be the possible reason behind the observed differences. Different MAPKs have been shown to regulate apoptosis in Jurkat cells (Kutuk et al., 2005; Pestka et al., 2005). rHP1286 failed to induce activation of any MAPK under the conditions tested, which could explain the reason behind absence of apoptosis in Jurkat cells. Currently, we are working on understanding the response of other cell types to HP1286 and the differences in the signaling pathways that are involved in the regulation of apoptosis. It was interesting to note the similarities and differences between HP1286 and JHP0290 (Li et al., 2012; Pathak et al., 2013; Tavares and Pathak, 2015). Studies have been initiated to identify the factors that regulate the similarities and differences between the host cell responses to JHP0290 and HP1286. Several strategies were adopted to confirm that the observed effect was not due to endotoxin contamination in the purified protein. Firstly, the purified protein was incubated with an endotoxin removal column to remove any LPS in the purified HP1286 and studies were performed using HEK293 cells (naturally lack receptors for endotoxin signaling) and HEK293-TLR4 cells (stably transfected with receptors for LPS signaling). rHP1286 was able to bind to both cell types, but failed to induce IL-8, thus confirming the endotoxin-independent effects. This conclusion was further supported by the observation that rHP1286 failed to induce significantly higher level of TNF in RAW264.7 cells and a mutant strain of *H. pylori* lacking HP1286 expression was significantly impaired in its ability to induce macrophage apoptosis.

Upon *H. pylori* challenge, macrophages produce TNF, a cytokine that is involved in pro- apoptotic signaling (Harris et al., 2000; Aggarwal, 2003; Pathak et al., 2013). We previously reported that another *H. pylori* secreted protein JHP0290 induces macrophage apoptosis via TNF-dependent and -independent pathways (Pathak et al., 2013). However, HP1286 appears to induce macrophage apoptosis via TNF-independent pathways since neutralizing antibodies against TNF had no effect on rHP1286-induced macrophage apoptosis. This view was further strengthened by the observation that rHP1286 failed to induce TNF in macrophages under the conditions, which induced macrophage apoptosis. ERK MAPK has been shown to regulate *H. pylori*-induced macrophage apoptosis (Asim et al., 2010). However, specific *H. pylori* virulence factor (s) that regulate macrophage apoptosis via ERK MAPK have not been identified so far. We recently identified *H. pylori* secreted protein JHP0290 as one of the regulators of ERK-dependent macrophage apoptosis (Pathak et al., 2013). In this study we identified another regulator of macrophage apoptosis in *H. pylori*. To our knowledge, HP1286 is only the second characterized virulence factor of *H. pylori* that induces macrophage apoptosis via ERK MAPK.

Sequence similarities suggest that HP1286 belongs to the YceI-like family of proteins (Sisinni et al., 2010). YceI-like family proteins have been suggested to play an important role in isoprenoid quinone metabolism or in transport or storage (Handa et al., 2005). However, 3D structure of the protein HP1286 revealed distinct properties from other members of the family including an inner cavity structural adaption for a new binding specificity and it was suggested that the physiological function of HP1286 might differ from other members of this family (Sisinni et al., 2010). It was also observed that under acidic stress, HP1286 was one of the limited numbers of proteins, which was overexpressed by a mutant strain of *H. pylori* (Ure1-negative) that was unable to incorporate urea (Toledo et al., 2002). This upregulation could be a bacterial strategy for survival in the hostile environment via either regulation of bacterial growth/survival or by affecting the responses of target host cells. Other reports have provided some information on the functional role of HP0243 (neutrophil-activating protein) and HP0485 (catalase-like enzyme), two other proteins that were found to be upregulated in Ure1-negative strain (Tomb et al., 1997; Tonello et al., 1999; Zanotti et al., 2002). Here, we have provided evidences that suggest the function of HP1286 during bacterial pathogenesis via regulation of macrophage apoptosis. In addition to regulations of host cell responses, HP1286 could also be involved in bacterial growth/survival since the structural features indicated that the function of the protein could be sequestering of fatty acids or amides in the environment of the bacterium, which may protect the external membrane from their surfactant properties and/or to affect bacterial metabolism by regulating the supply of necessary fatty acids (Sisinni et al., 2010). However, we did not observe any difference in the bacterial growth or viability in Wt and HP1286 protein deficient strains of *H. pylori* 26695. Further studies are required to understand the role of HP1286 in bacterial growth or survival, if any.

During persistent *H. pylori* infection, the number of macrophage gradually declines, which is likely to occur via induction of apoptosis (Asim et al., 2010). This decline is expected to protect the bacteria from the microbicidal activities of macrophages, which in turn will help in persistence. Therefore, by virtue of its ability to induce macrophage apoptosis, HP1286 could possibly play an important role in the bacterial persistence and may also influence subsequent disease development.

## Author contributions

RT and SP conceived and designed the experiments, performed the experiments, analyzed data, and wrote the manuscript.

## Funding

This work was supported by the Swedish Research Council, Åke Wiberg Foundation and Carl Tryggers Foundation. The funders had no role in the study design, data collection, and analysis, decision to publish, or preparation of the manuscript.

### Conflict of interest statement

The authors declare that the research was conducted in the absence of any commercial or financial relationships that could be construed as a potential conflict of interest.
